# 

**Published:** 1886-12

**Authors:** 


SPECIAL.—*Hereafter this* JOURNAL *will be issued punctually on the first of every month, instead of the fifteenth, as heretofore.*

### BOOK NOTICES.

The December "Century" is so replete of good things that it is quite out of the question to mention them all. The life of Abraham Lincoln, by his former war-time Secretaries, increases in interest as it progresses, and is sure to become standard.

The Second Day at Gettysburg, by General Hunt, Chief of Artillery of the Army of the Potomac, is a valuable contribution to our Civil War history.

Ashland, the home of Henry Clay, is an admirable contribution, and the large engraved likeness of Kentucky's most accomplished statesman, which prefaces it, is drawn to the life. All in all, the "Century" is perhaps to-day the leading monthly of our time, and well deserves its immense circulation.

"Irene; or The Road to Freedom," by Sada Bailey Fowler.

This was evidently intended for an advanced novel, for none of the accepted ideas of medicine, morals or politics are mentioned, except for the purpose of being ridiculed; while theories are proposed which may be in vogue a century hence, but are not so now. The plot is well laid, and the story, where confined to it, well told; but the writer goes out of her way to attack every grievance of her sex, whether fancied or real, in a most quixotic and amusing fashion. Starting out with a moderate statement of women's rights, the author concludes by drawing as a logical conclusion thereto a sort of social anarchism. A continuation is promised to appear shortly. Published by H. N. Fowler, 1123 Arch St., Philadelphia.

